# One-Year Outcomes of Phacoemulsification Combined with Trabecular Micro-Bypass Stent Implantation and Goniosynechialysis in Primary Angle Closure Glaucoma

**DOI:** 10.3390/jcm15145670

**Published:** 2026-07-20

**Authors:** Yu-Jin Choi, Chungkwon Yoo, Eun Jung Jung

**Affiliations:** Department of Ophthalmology, Korea University College of Medicine, 73 Goryeodae-ro, Seongbuk-gu, Seoul 02841, Republic of Korea; qwe123qwerhhh@gmail.com (Y.-J.C.); cleanerg@naver.com (E.J.J.)

**Keywords:** primary angle closure glaucoma, trabecular micro-bypass stent, phacoemulsification, intraocular pressure, minimally invasive glaucoma surgery

## Abstract

**Background/Objectives:** Trabecular micro-bypass (iStent) implantation has been shown to effectively reduce intraocular pressure (IOP) and medication burden in open-angle glaucoma. However, its efficacy in primary angle closure glaucoma (PACG) remains unclear. We conducted this study to evaluate the one-year clinical outcomes of phacoemulsification combined with goniosynechialysis and iStent implantation (Phaco-GSL-iStent) in Korean patients with PACG. **Methods:** This retrospective study included 65 PACG eyes that underwent Phaco-GSL-iStent with follow-up of ≥12 months. Postoperative changes in mean IOP and glaucoma medications were assessed through postoperative 12 months. Predictors of postoperative changes in IOP and glaucoma medications were also evaluated. **Results:** Mean IOP significantly decreased from 18.3 ± 0.4 to 13.8 ± 0.3 mmHg at postoperative 12 months (*p* < 0.001). Mean number of glaucoma medications showed a statistically significant but small decrease from 2.85 ± 0.15 to 2.44 ± 0.13 at 12 months (*p* = 0.008). The 12-month cumulative qualified success rate was 86.2%. In multivariable analysis, IOP reduction at postoperative 12 months was positively associated with preoperative IOP (B = 0.707, *p* < 0.001). IOP spikes (defined as an increase of 5 mmHg or more from the baseline) were observed in two eyes (3.1%). **Conclusions:** These one-year outcomes suggest that Phaco-GSL-iStent can be an effective option to lower IOP in patients with PACG. However, the reduction in glaucoma medication use was limited in this study sample.

## 1. Introduction

Primary angle closure glaucoma (PACG) is characterized by appositional or synechial closure of the iridotrabecular angle, leading to impaired aqueous outflow, elevated intraocular pressure (IOP), and progressive optic neuropathy. In eyes with angle closure, anterior segment anatomy, particularly a shallow anterior chamber and a relatively anteriorly positioned and/or thickened crystalline lens, results in an anatomically crowded anterior segment in which the peripheral iris obstructs the trabecular meshwork or forms synechiae, thereby blocking aqueous outflow [1,2,3].

As with primary open-angle glaucoma (POAG), reduction of elevated IOP is the only proven treatment to prevent glaucomatous progression of PACG. However, one also needs to consider interventions (laser iridotomy or cataract extraction) to prevent further narrowing of the anterior chamber and the development of pupillary block and/or to widen the anterior chamber angle when managing PACG.

Phacoemulsification has been well established to effectively restore vision and reduce IOP in eyes with cataract and PACG [4,5,6]. Its IOP-lowering effect has been greater in eyes with PACG compared to POAG [7,8,9]. A prior randomized controlled trial in Hong Kong demonstrated that in PACG eyes without visually significant cataract, phacoemulsification achieved a 34% reduction in IOP, which was comparable to the 36% reduction observed with trabeculectomy. Notably, phacoemulsification showed a better safety profile, with a 4% complication rate compared to 46% in the trabeculectomy group [10]. Furthermore, the EAGLE trial provided evidence that phacoemulsification can be a good alternative to laser peripheral iridotomy (LPI) even in primary angle closure patients without visually significant cataract [11].

Despite the angle-widening effects of phacoemulsification, peripheral anterior synechiae (PAS) may persist after the surgery and still contribute to residual outflow obstruction. Goniosynechialysis (GSL) has therefore been used as an adjunctive procedure to mechanically separate PAS from the trabecular meshwork, thereby allowing aqueous humor access to the re-exposed trabecular meshwork [12,13]. This additional IOP-lowering effect has been suggested to be greater in eyes with fresh PAS than in those with old PAS. However, some studies have reported that the addition of GSL to phacoemulsification may not provide a meaningful additional reduction in IOP or glaucoma medication burden compared with phacoemulsification alone in PACG eyes [14,15]. Such lack of IOP reduction may be attributed to the irreversible dysfunction of the trabecular meshwork and downstream outflow pathways from long-standing PAS in PACG eyes. Although GSL addresses the pretrabecular component by releasing PAS, it does not directly reverse trabecular and distal outflow dysfunction. In fact, chronic mechanical apposition can lead to structural changes, including fibrosis, that may be partially irreversible, rendering simple anatomical opening insufficient [14,16]. Therefore, residual trabecular outflow impairment may persist even after anatomic angle opening, which supports the need for trabecular-targeting approaches [17,18]. Given these pathophysiological limitations, cutting or stenting the trabecular meshwork combined with phacoemulsification and GSL (Phaco-GSL) may re-establish the aqueous humor communication between the anterior chamber and the post-trabecular meshwork pathway and enhance the conventional outflow in PACG eyes.

Recently, there have been some reports that introduced the beneficial role of angle-targeting minimally invasive glaucoma surgery (MIGS) combined with phacoemulsification in PACG patients. Several studies from China have reported the effective IOP reduction of goniotomy combined with Phaco-GSL in PACG patients [19,20,21,22]. Although trabecular micro-bypass stent (iStent) implantation combined with Phaco-GSL has also been reported to provide similar IOP reduction, there are relatively few studies on its clinical outcomes in Asian PACG patients [18,23].

Thus, we conducted the present study to evaluate the one-year clinical outcomes of phacoemulsification combined with iStent implantation and GSL (Phaco-GSL-iStent) in Korean patients with PACG.

## 2. Materials and Methods

### 2.1. Study Design and Criteria

This retrospective study included patients diagnosed with PACG who underwent trabecular micro-bypass stent (iStent, Glaukos Corp., San Clemente, CA, USA) implantation combined with phacoemulsification and GSL at the Department of Ophthalmology, Korea University Anam Hospital, between April 2020 and March 2025 and who had been followed for at least 12 months postoperatively. The study was approved by the Institutional Review Board (IRB) of Korea University Medical Center (approval number: 2024AN0339) and adhered to the principles of the Declaration of Helsinki. All clinical data were de-identified before statistical analysis, and the requirement for written informed consent was waived by the IRB due to the retrospective nature of the study.

PACG was defined according to the International Society for Geographical and Epidemiological Ophthalmology (ISGEO) criteria proposed by Foster et al. [1]: (1) anterior chamber angle with the posterior trabecular meshwork not visible for ≥180° on gonioscopy; (2) elevated IOP (IOP > 21 mmHg) and/or PAS; and (3) glaucomatous optic neuropathy with corresponding visual field defects.

The inclusion criteria were as follows: (1) age ≥ 40 years; (2) diagnosis of PACG based on the ISGEO criteria; (3) PAS > 90° of the entire angle on gonioscopy; (4) presence of a visually significant cataract; (5) history of Phaco-GSL-iStent surgery; and (6) availability of postoperative follow-up clinical data for at least 12 months.

The exclusion criteria were as follows: (1) corneal diseases or a history of refractive surgery that could compromise the accuracy of IOP measurement; (2) ocular or systemic diseases that could affect the interpretation of visual acuity or visual field test results; (3) intraoperative complications including posterior capsular rupture, vitreous loss, or nucleus drop; and (4) any secondary cause of ACG (e.g., neovascularization, pseudoexfoliation, trauma, uveitis, etc.).

During the study period, 72 eyes of 57 patients underwent phacoemulsification combined with GSL and iStent implantation. Among them, 7 eyes were excluded because of insufficient postoperative follow-up. Accordingly, a total of 65 eyes from 50 patients met the predefined inclusion criteria and were included in the final analysis.

### 2.2. Surgical Technique and Postoperative Management

All Phaco-GSL-iStent procedures were performed by a single glaucoma specialist (C.Y.). Briefly, topical anesthesia was administered using 0.5% proparacaine or a 2% lidocaine-epinephrine mixture. Uncomplicated cataract surgery was completed through a 2.75 mm corneal incision, and a single-piece hydrophilic intraocular lens was implanted in the capsular bag. Under gonioscopic visualization using a Swan-Jacob gonioprism (Ocular Instruments, Inc., Bellevue, WA, USA), the nasal 180° of the angle was examined. GSL was performed using a Sinskey hook (Katena Products, Inc., Denville, NJ, USA) if any PAS was present in the nasal 180° of the entire anterior chamber angle. Then, the iStent devices were implanted into the trabecular meshwork at least two clock hours apart. After the viscoelastic was completely removed using the automated irrigation and aspiration system, the corneal incision was closed watertight with a single 10-0 nylon suture.

The iStent devices used in this study included iStent, iStent inject, iStent inject W, or iStent infinite (Glaukos Corporation, Aliso Viejo, CA, USA). The first-generation iStent is a snorkel-shaped device made of heparin-coated titanium that is implanted in Schlemm’s canal using a preloaded injector. Each injector is preloaded with one stent. The second-generation, iStent inject, is a bullet-shaped device with a head, a thorax and a flange that is placed in the Schlemm’s canal, trabecular meshwork, and anterior chamber, respectively. The inserter is preloaded with 2 stents. The third-generation, iStent inject W, is similar to the iStent inject; however, the only difference is that it has a wider flange, which was designed to prevent over-implantation into the trabecular meshwork. The fourth-generation, iStent infinite, consists of 3 iStent inject W stents in a single preloaded injector. Overall, the first-generation iStent has a greater diameter (120 μm vs. 80 μm) than the subsequent-generation stents. The number of stents implanted per eye was greater for iStent infinite cases than for prior-generation ones (3 vs. 2) [24,25].

Postoperatively, topical 0.5% moxifloxacin and 1% prednisolone were administered four times daily, and 0.1% bromfenac was instilled twice daily for 1 month. The topical steroid use was extended beyond 4 weeks depending on the severity of intraocular inflammation; notably, it was tapered more slowly or switched to a weaker one (0.5% loteprednol) over 6 to 12 weeks in eyes with prior peripheral iridotomy. Glaucoma eyedrops were partially reduced or re-adjusted, on an individual basis, at the surgeon’s discretion according to the target IOP, severity/progression of glaucoma or tolerance of glaucoma eyedrops.

### 2.3. Data Collection and Outcome Measures

Baseline demographic and clinical data, as well as postoperative outcomes, were retrospectively collected from the medical records. Baseline data included systemic medical history, prior glaucoma laser treatments, history of antiglaucoma medication use, history of previous intraocular surgeries, as well as data obtained from preoperative ophthalmic examinations, including best-corrected visual acuity (BCVA), biometry using the IOLMaster 700 (Carl Zeiss Meditec AG, Jena, Germany), IOP measured with Goldmann applanation tonometry, slit-lamp examination, and fundus examination. Gonioscopic findings, obtained during routine clinical examinations in a dark room using a 4-mirror Posner goniolens (Ocular Instruments Inc., Bellevue, WA, USA), were retrospectively reviewed from medical records. The circumferential extent of PAS was categorized into two groups: Group 1, 90° < PAS ≤ 180°; Group 2, PAS > 180°. Baseline imaging and functional data included peripapillary retinal nerve fiber layer (RNFL) imaging using optical coherence tomography (Triton DRI OCT; Topcon Corporation, Tokyo, Japan) and visual field testing using the Humphrey Field Analyzer (Carl Zeiss Meditec Inc., Dublin, CA, USA).

Postoperative routine follow-up evaluations included IOP measurement and slit-lamp biomicroscopy. For the present study, the data recorded at 1 day, 1 week, 1 month, 3 months, 6 months, and 12 months after surgery were extracted from the medical records for analysis.

The primary outcomes were the changes in IOP, the changes in the number of antiglaucoma medications, and the surgical success rate at postoperative 12 months. Complete surgical success was defined as achieving a ≥20% reduction in IOP from baseline and maintaining an absolute IOP of ≤15 mmHg postoperatively without glaucoma medications. Qualified surgical success was defined as achieving the same IOP criteria with or without glaucoma medications. Qualified surgical success additionally required no increase in the number of antiglaucoma medications compared with baseline. For both definitions, surgical success required no need for additional laser or incisional glaucoma surgery, no loss of light perception, and the absence of hypotony. Surgical failure was defined as failure to meet the corresponding criteria at two consecutive postoperative visits or the need for additional glaucoma surgery [26,27,28,29,30].

Safety indicators included the occurrence of intraoperative and postoperative complications, the need for additional glaucoma surgery, and the presence of an IOP spike. IOP spikes were classified into four types according to the criteria described by Bojikian et al. [31]: an increase of ≥50% from the preoperative IOP (Spike 1); an absolute IOP ≥ 30 mmHg after surgery (Spike 2); an increase of ≥10 mmHg from baseline (Spike 3); or an increase of ≥5 mmHg from baseline (Spike 4). IOP spikes were evaluated at postoperative day 1, week 1, and month 1, respectively.

Additionally, the associations of the baseline predictive factors with postoperative changes in IOP or the number of glaucoma medications at 12 months were evaluated.

### 2.4. Statistical Analysis

Statistical analyses were performed using SPSS software (version 18.0; SPSS Inc., Chicago, IL, USA). The sample size was determined by the number of eligible patients available during the study period, and all consecutive eligible patients were included. A sensitivity power analysis was conducted using G*Power (version 3.1) to estimate the minimum detectable effect size. Continuous variables were presented using the mean and standard deviation, and categorical variables were expressed as percentages. For analyses of overall surgical outcomes, including longitudinal changes in IOP, number of glaucoma medications, and survival analyses, all eligible eyes were included to describe the postoperative clinical outcomes of the full eye-level cohort. Paired t-tests were conducted to evaluate significant changes in mean IOP and the number of medications at baseline and at 1 day, 1 week, 1 month, 3 months, 6 months, and 12 months postoperatively. Bonferroni correction was applied for multiple comparisons, and a corrected significance threshold of *p* < 0.0083 (0.05/6) was used.

For predictor analyses, including linear regression analyses, a one-eye-per-patient dataset was used to avoid potential inter-eye correlation and to satisfy the independence assumption. In patients in whom both eyes were eligible, the first-operated eye was selected for inclusion in the predictor analyses. Univariable linear regression analyses were first performed to evaluate factors associated with the IOP reduction and the reduction in the number of glaucoma medications at 12 months postoperatively. To reduce the risk of overfitting due to the limited sample size, multivariable linear regression analyses were performed using a reduced number of covariates selected based on clinical relevance and the results of univariable analyses, rather than including all candidate variables simultaneously. For the IOP reduction at 12 months, the reduced multivariable model included baseline IOP and clinically relevant covariates, including age, extent of PAS, and anterior chamber depth. For medication reduction at 12 months, the reduced multivariable model included the baseline number of glaucoma medications, anterior chamber depth, extent of PAS, and visual field index. Model diagnostics were assessed for the multivariable regression models. Multicollinearity was evaluated using variance inflation factors, and residual plots were reviewed to assess linearity and homoscedasticity.

Surgical success was assessed using Kaplan–Meier survival analysis. *p* < 0.05 was considered statistically significant.

## 3. Results

### 3.1. Baseline Characteristics

A total of 65 eyes from 50 patients were included in the study. Baseline demographic and clinical characteristics of the study participants are presented in Table 1. Prior LPI history was present in 52.3% of the eyes. Regarding the extent of PAS, 81.5% had PAS involvement between 90° and 180° of the angle, and 18.5% had involvement greater than 180°. The proportions of eyes with early, moderate, and severe glaucoma were 31%, 29%, and 40%, respectively. Table 1 also lists the proportions of types of the trabecular micro-bypass stents implanted. Fifty-one percent of the study eyes had iStent inject W; 32%, 14% and 3% had iStent, iStent infinite and iStent inject, respectively. Two stents were implanted per eye for iStent, iStent inject or iStent inject W, whereas 3 stents were implanted per eye for iStent infinite.

### 3.2. Changes in IOP and Number of Glaucoma Medications

**Figure 1:**
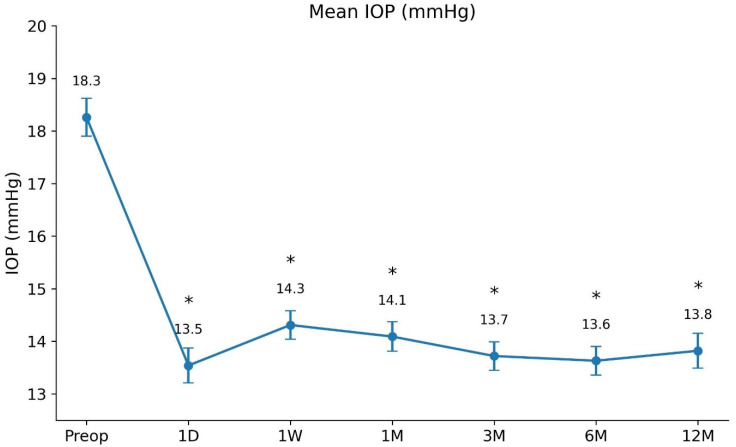
Mean intraocular pressure (IOP) changes during the 12-month follow-up period after phacoemulsification combined with goniosynechialysis and iStent implantation in the overall study population. Error bars indicate the standard error of the mean. Asterisks indicate statistically significant differences from baseline using paired *t*-tests with Bonferroni correction for six comparisons (* *p* < 0.0083).

In the overall study population, the mean IOP decreased significantly from 18.3 ± 0.4 mmHg preoperatively to 13.5 ± 0.3 mmHg at 1 day postoperatively (*p* < 0.001). Significant reductions from the baseline were also observed at 1 week (14.3 ± 0.3 mmHg), 1 month (14.1 ± 0.3 mmHg), 3 months (13.7 ± 0.3 mmHg), 6 months (13.6 ± 0.3 mmHg), and 12 months (13.8 ± 0.3 mmHg), all *p* < 0.001 (Figure 1).

**Figure 2:**
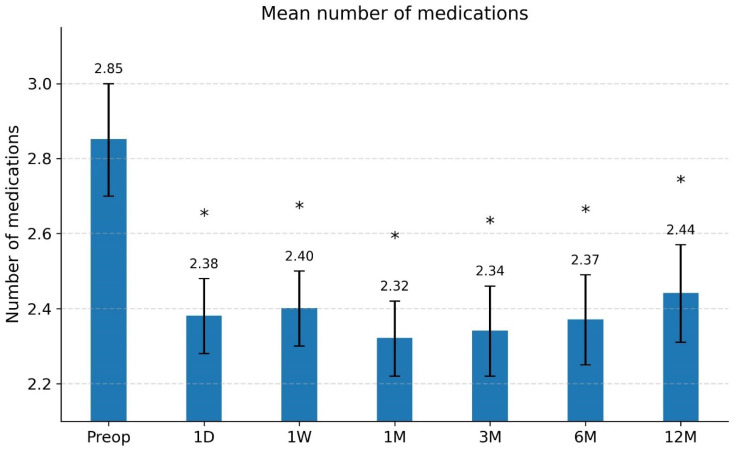
Changes in the mean number of glaucoma medications during the 12-month follow-up period after phacoemulsification combined with goniosynechialysis and iStent implantation in the overall study population. Error bars indicate the standard error of the mean. Asterisks indicate statistically significant differences from baseline using paired t-tests with Bonferroni correction for six comparisons (* *p* < 0.0083).

In the overall study population, the mean number of antiglaucoma medications decreased from 2.85 ± 0.15 at baseline to 2.38 ± 0.10 at 1 day postoperatively (*p* < 0.001). Significant reductions from baseline were also observed at 1 week (2.40 ± 0.10, *p* < 0.001), 1 month (2.32 ± 0.10, *p* < 0.001), 3 months (2.34 ± 0.12, *p* < 0.001), 6 months (2.37 ± 0.12, *p* < 0.001), and 12 months (2.44 ± 0.13, *p* = 0.008) (Figure 2).

**Figure 3:**
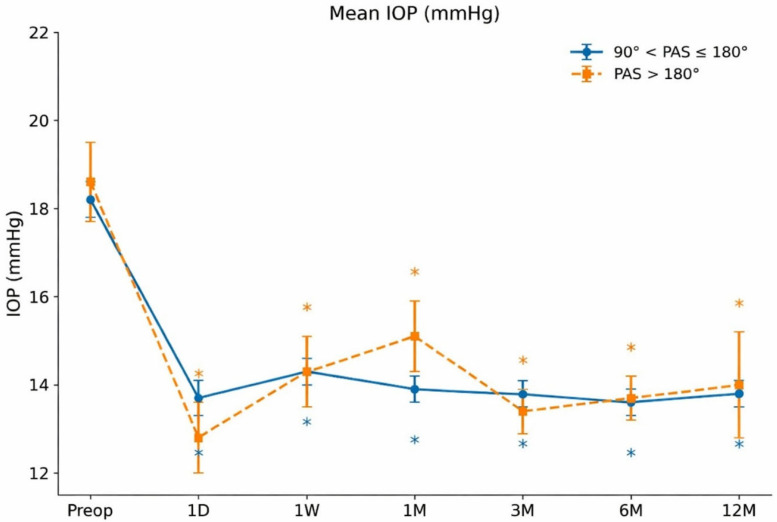
Mean intraocular pressure (IOP) changes during the 12-month follow-up period after phacoemulsification combined with goniosynechialysis and iStent implantation, stratified by peripheral anterior synechiae (PAS) extent (90° < PAS ≤ 180° vs. PAS > 180°). Error bars indicate the standard error of the mean. Asterisks indicate statistically significant differences from baseline within each PAS subgroup using paired *t*-tests with Bonferroni correction for six comparisons (* *p* < 0.0083).

**Figure 4:**
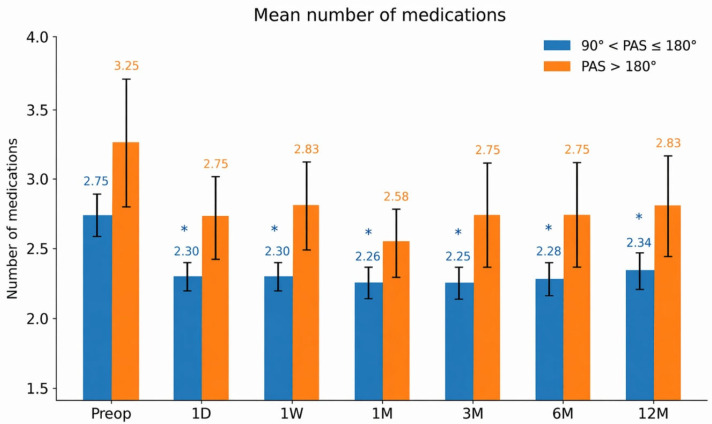
Changes in the mean number of glaucoma medications during the 12-month follow-up period after phacoemulsification combined with goniosynechialysis and iStent implantation, stratified by peripheral anterior synechiae (PAS) extent (90° < PAS ≤ 180° vs. PAS > 180°). Error bars indicate the standard error of the mean. Asterisks indicate statistically significant differences from baseline within each PAS subgroup using paired t-tests with Bonferroni correction for six comparisons (* *p* < 0.0083).

When stratified by the PAS extent, the eyes with PAS between 90° and 180° showed a significant decrease in the mean IOP from 18.2 ± 0.4 mmHg preoperatively to 13.7 ± 0.4 mmHg at 1 day postoperatively (*p* < 0.001). Significant reductions from baseline were also observed at 1 week (14.3 ± 0.3 mmHg), 1 month (13.9 ± 0.3 mmHg), 3 months (13.8 ± 0.3 mmHg), 6 months (13.6 ± 0.3 mmHg), and 12 months (13.8 ± 0.3 mmHg) (all *p* < 0.001). In the eyes with PAS between 90° and 180°, the mean number of antiglaucoma medications decreased from 2.75 ± 0.15 at baseline to 2.30 ± 0.10 at 1 day postoperatively (*p* < 0.001). Significant reductions from baseline were also observed at 1 week (2.30 ± 0.10, *p* < 0.001), 1 month (2.26 ± 0.10, *p* < 0.001), 3 months (2.25 ± 0.11, *p* < 0.001), 6 months (2.28 ± 0.12, *p* < 0.001), and 12 months (2.34 ± 0.14, *p* = 0.004) (Figure 3 and Figure 4).

In the eyes with PAS > 180°, the mean IOP decreased significantly at postoperative day 1 (18.6 ± 0.9 mmHg to 12.8 ± 0.8 mmHg, *p* = 0.001). Significant reductions from baseline were also observed at 1 week (14.3 ± 0.8 mmHg, *p* = 0.006), 1 month (15.1 ± 0.8 mmHg, *p* = 0.001), 3 months (13.4 ± 0.5 mmHg, *p* < 0.001), 6 months (13.7 ± 0.5 mmHg, *p* = 0.001), and 12 months (14.0 ± 1.2 mmHg, *p* = 0.003). In these eyes, the mean number of antiglaucoma medications did not differ significantly between the baseline and each time point (Figure 3 and Figure 4).

**Figure 5:**
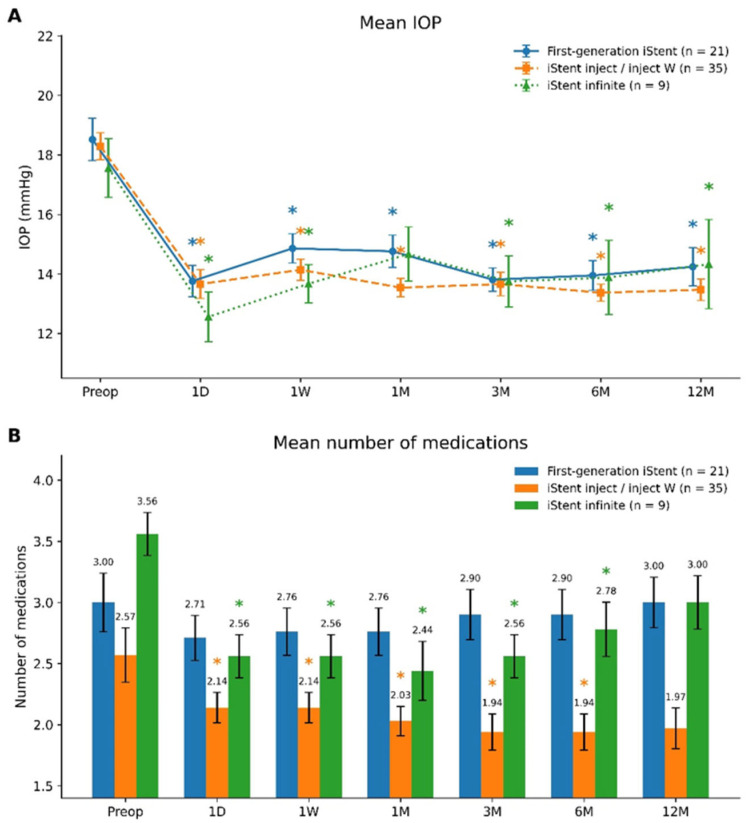
Changes in mean intraocular pressure (IOP) and the number of glaucoma medications after phacoemulsification combined with goniosynechialysis and iStent implantation according to the type of implanted stent. (**A**) Mean IOP. (**B**) Mean number of glaucoma medications. Error bars indicate the standard error of the mean. Asterisks indicate statistically significant differences from baseline within each device subgroup using paired *t*-tests with Bonferroni correction for six comparisons (* *p* < 0.0083).

When stratified by the stent type, first-generation iStent eyes (*n* = 21) showed significant IOP reductions at all postoperative visits, from 18.5 ± 0.7 mmHg preoperatively to 14.2 ± 0.6 mmHg at 12 months (all *p* < 0.001). Medication use decreased transiently but was not significantly different from baseline at any visit, including 12 months (3.00 ± 0.21, *p* = 1.000) (Figure 5).

Because only two eyes received the iStent inject and its intratrabecular design is identical to that of the iStent inject W, these eyes were combined with the iStent inject W group for analysis. In the eyes with the iStent inject or iStent inject W implanted (*n* = 35), the mean IOP decreased significantly from 18.3 ± 0.5 mmHg preoperatively to 13.5 ± 0.4 mmHg at 12 months, with significant reductions at all postoperative visits (all *p* < 0.001). Mean medication use decreased significantly through 6 months, from 2.57 ± 0.22 at baseline to 1.94 ± 0.15 at 6 months (*p* = 0.003), but the reduction was not significant at 12 months (1.97 ± 0.17, *p* = 0.010) (Figure 5).

**Figure 6:**
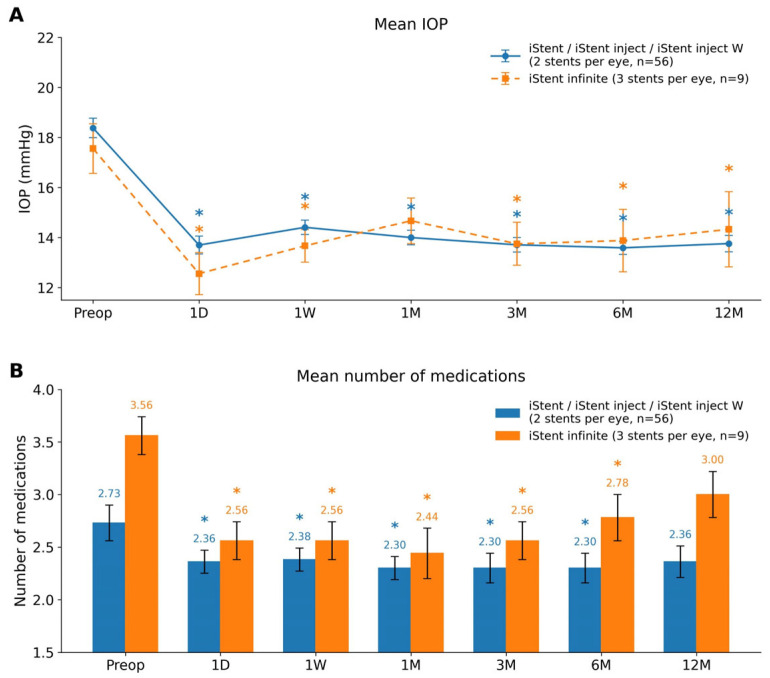
Changes in mean intraocular pressure (IOP) and number of glaucoma medications after phacoemulsification combined with goniosynechialysis and iStent implantation according to the number of stents implanted per eye. (**A**) Mean IOP. (**B**) Mean number of glaucoma medications. Error bars indicate the standard error of the mean. Asterisks indicate significant differences from baseline within each subgroup using paired *t*-tests with Bonferroni correction for six comparisons (* *p* < 0.0083).

In the iStent infinite group (*n* = 9), in which three stents were implanted per eye, IOP decreased significantly from 17.6 ± 1.0 mmHg preoperatively to 14.3 ± 1.5 mmHg at 12 months (*p* = 0.003), with significant reductions at all visits except at 1 month (*p* = 0.010). Medication use was significantly reduced through 6 months, but not at 12 months (3.00 ± 0.22, *p* = 0.047) (Figure 5 and Figure 6).

We also assessed the IOP and medication burden outcomes in the two-stent-per-eye group (first-generation iStent, iStent inject, or iStent inject W, *n* = 56). In this two-stent group, the mean IOP decreased significantly from 18.4 ± 0.4 mmHg preoperatively to 13.8 ± 0.3 mmHg at 12 months, with significant reductions at all postoperative visits (all *p* < 0.001). Mean medication use decreased significantly through 6 months, from 2.73 ± 0.17 at baseline to 2.30 ± 0.14 at 6 months (*p* = 0.004), but was not significantly reduced at 12 months (2.36 ± 0.15, *p* = 0.029) (Figure 6).

In the stent-type-stratified linear mixed-effects model, IOP changed significantly over time after surgery (F = 37.508, *p* < 0.001), whereas neither stent group (F = 2.017, *p* = 0.144) nor the time-by-group interaction (F = 0.753, *p* = 0.699) was significant. Thus, longitudinal IOP changes did not differ among the iStent, iStent inject/inject W, and iStent infinite groups. By contrast, medication use varied significantly by stent type, with significant effects of time (F = 11.390, *p* < 0.001), group (F = 5.051, *p* = 0.010), and the time-by-group interaction (F = 2.878, *p* = 0.001). At baseline, the iStent infinite group used more medications than the iStent inject/inject W group (*p* = 0.018). Postoperatively, Bonferroni-adjusted comparisons showed greater medication use in the iStent group than in the iStent inject/inject W group at 1, 3, 6, and 12 months (*p* = 0.047, 0.003, 0.003, and 0.002, respectively), with no significant postoperative differences involving the iStent infinite group.

In the analysis stratified by the number of implanted stents, IOP changed significantly over time after surgery (F = 20.957, *p* < 0.001), but neither stent number (F = 0.240, *p* = 0.625) nor the time-by-stent-number interaction (F = 0.598, *p* = 0.732) were significant. This indicates that longitudinal IOP changes were similar between eyes receiving two and three stents. In contrast, medication use changed differently between the two groups, with significant effects of time (F = 15.628, *p* < 0.001) and the time-by-stent-number interaction (F = 3.541, *p* = 0.002), although the main effect of stent number was not significant (F = 1.050, *p* = 0.310).

Regarding visual and refractive outcomes, the mean BCVA improved from 0.41 ± 0.40 logMAR preoperatively to 0.20 ± 0.23 logMAR at 12 months postoperatively (*p* < 0.001). The mean spherical equivalent changed from 0.70 ± 1.57 D preoperatively to 0.36 ± 1.02 D at 12 months postoperatively; however, this change was not statistically significant (*p* = 0.131).

### 3.3. Surgical Success and Survival Analysis

**Figure 7:**
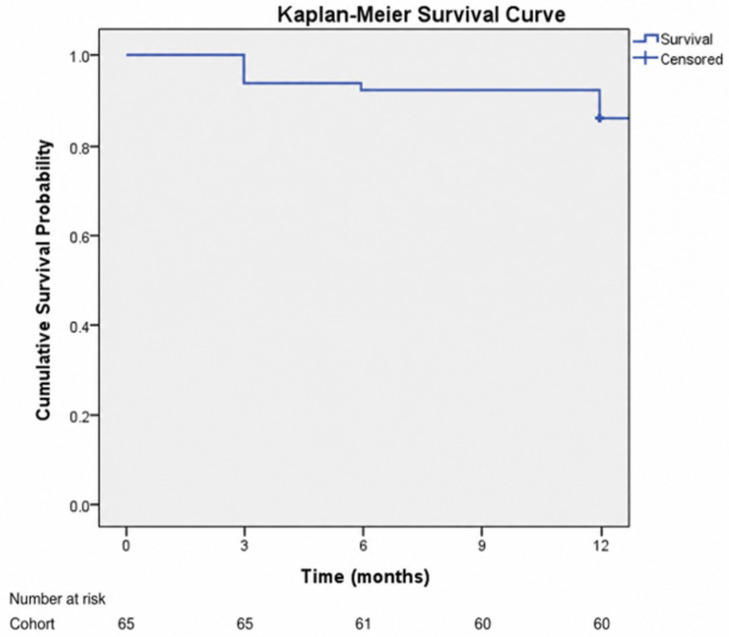
Kaplan–Meier survival curve for qualified surgical success after phacoemulsification combined with goniosynechialysis and iStent implantation.

Kaplan–Meier survival analysis was performed to estimate cumulative surgical success over time. At 12 months postoperatively, the cumulative probability of qualified surgical success was 86.2% (95% CI, 75.1–92.6%) (Figure 7). No eye achieved complete surgical success at 12 months because all eyes required glaucoma medications postoperatively.

### 3.4. Postoperative Complications and Additional Glaucoma Surgery

Postoperative IOP spikes were observed in a small number of eyes across the different spike definitions. Among the 65 eyes, on postoperative day 1, Spike 4 (a ≥5 mmHg increase from baseline IOP) was observed in one eye (1.5%), with no cases of Spike 1 (a ≥50% increase from baseline IOP), Spike 2 (IOP ≥ 30 mmHg), or Spike 3 (a ≥10 mmHg increase from baseline IOP). This eye was managed by adding topical brimonidine twice daily, and the IOP subsequently improved and remained stable by postoperative day 5. At week 1, Spike 4 was observed in another eye (1.5%), with no cases of Spike 1, Spike 2, or Spike 3. The IOP improved without any additional medication, remaining stable thereafter. At postoperative month 1, no IOP spike was observed under any of these IOP spike definitions.

Microhyphema was observed in 3 eyes (4.6%), all of which were mild and transient, and no vision-threatening complications occurred. Additional glaucoma surgery was required in 1 eye (1.5%). This eye showed IOP elevation at 1 year postoperatively, which seems to have occurred due to the intake of steroid-containing oral medications. Despite maximal medical IOP-lowering therapy, the IOP remained uncontrolled, thereby necessitating a subsequent filtering surgery, i.e., implantation of an A-Stream Glaucoma Shunt (Microt Inc., Seoul, Republic of Korea).

### 3.5. IOP Changes and Associated Factors

Using the one-eye-per-patient dataset, univariable linear regression analysis showed that preoperative IOP had a significant positive association with the IOP reduction at 12 months postoperatively (B = 0.672; 95% CI, 0.442 to 0.903; *p* < 0.001). Other preoperative variables, including age, axial length, white-to-white distance, anterior chamber depth, central corneal thickness, extent of PAS, RNFL thickness, number of glaucoma medications, lens thickness, and visual field indices, did not show significant associations (Table 2).

In the reduced multivariable model including age, ACD, extent of PAS, and preoperative IOP, only preoperative IOP remained significantly associated with IOP reduction at postoperative 12 months (B = 0.707; 95% CI, 0.443 to 0.970; *p* < 0.001). No substantial multicollinearity was observed, with variance inflation factors ranging from 1.011 to 1.100 (Table 2).

### 3.6. Postoperative Medication Changes and Associated Factors

Using the one-eye-per-patient dataset, univariable linear regression analysis showed that the number of preoperative glaucoma medications, ACD, extent of PAS, and VFI were significantly associated with the changes in the number of medications at 12 months postoperatively. The number of preoperative glaucoma medications showed a significant positive regression coefficient (B = 0.574; 95% CI, 0.375 to 0.773; *p* < 0.001), whereas ACD and extent of PAS showed significant negative regression coefficients (ACD: B = −1.066; 95% CI, −2.076 to −0.056; *p* = 0.039; extent of PAS: B = −0.141; 95% CI, −0.268 to −0.014; *p* = 0.030). VFI also showed a significant positive regression coefficient (B = 0.013; 95% CI, 0.001 to 0.026; *p* = 0.038). Other variables, including age, axial length, white-to-white distance, central corneal thickness, RNFL thickness, preoperative IOP, lens thickness, MD, and PSD, did not show statistically significant associations with the change in the number of medications at 12 months (Table 3).

In the reduced multivariable model including the number of preoperative glaucoma medications, ACD, extent of PAS, and VFI, all four variables remained significantly associated with medication reduction at 12 months. The number of preoperative glaucoma medications and VFI showed significant positive regression coefficients (number of medications: B = 0.695; 95% CI, 0.545 to 0.845; *p* < 0.001; VFI: B = 0.014; 95% CI, 0.007 to 0.022; *p* = 0.001). In contrast, ACD and extent of PAS showed significant negative regression coefficients (ACD: B = −1.150; 95% CI, −1.805 to −0.496; *p* = 0.001; extent of PAS: B = −0.122; 95% CI, −0.201 to −0.043; *p* = 0.003). No substantial multicollinearity was observed, with variance inflation factors ranging from 1.056 to 1.159 (Table 3).

## 4. Discussion

### 4.1. Main Postoperative Outcomes and Comparison with Previous Studies

The present study found that iStent implantation combined with phacoemulsification and GSL effectively decreased IOP and reduced the number of glaucoma medications in patients with PACG up to 1 year after surgery. This finding is in line with earlier studies of angle-targeted MIGS performed in combination with cataract surgery in PACG patients [32,33,34].

In PACG, restoration of an open-angle configuration does not always translate into proportional long-term IOP reduction [35]. The PAS may represent not only a reversible site of iridotrabecular obstruction but also a marker of chronic or repetitive iridotrabecular contact, during which the trabecular meshwork and the conventional outflow pathway can undergo structural and functional compromise. Accordingly, even after GSL and apparent gonioscopic angle opening, residual outflow resistance may persist [36]. Moreover, postoperative inflammation and wound healing responses after mechanical synechialysis may lead to reformation of PAS. Based on this hypothetical mechanistic rationale, we combined phacoemulsification with GSL to open the angle and facilitate adequate access for device placement, and we also added iStent implantation to bypass trabecular meshwork resistance and promote aqueous outflow through Schlemm’s canal in the anatomically accessible regions.

Consistent with our observations, prior studies have reported meaningful IOP reduction with a combined surgical approach of phacoemulsification, GSL, and goniotomy in eyes with PACG. In a multicenter observational study by Song et al. [21] of 83 eyes with advanced PACG and cataract undergoing this combined procedure, mean IOP decreased from 27.4 ± 7.3 mmHg at baseline to 14.2 ± 2.6 mmHg at 12 months, and the mean number of glaucoma medications decreased from 2.0 to 0.3. The complete and qualified success rates at 1 year were 89.1% and 95.2%, respectively. In another retrospective observational study of medically uncontrolled advanced PACG, Zhang et al. [19] reported that mean IOP decreased from 27.6 ± 11.4 mmHg preoperatively to 16.5 ± 4.0 mmHg at 12 months after phacoemulsification combined with GSL and goniotomy. In addition, Shokoohi-Rad and colleagues [22] compared phacoemulsification with visco-goniosynechialysis plus goniotomy versus phacoemulsification with visco-goniosynechialysis alone in 63 eyes and reported that the mean IOP reduction at 9-month follow-up was greater (6.9 mmHg vs. 4.6 mmHg) with phacoemulsification combined with visco-goniosynechialysis and goniotomy.

Recently, several studies have reported clinical outcomes of phacoemulsification combined with iStent implantation, performed with or without GSL, in PACG. Hernstadt and coworkers [32] reported 1-year outcomes of combined phacoemulsification and iStent implantation in eyes with primary angle-closure disease, including PAC and PACG. Mean IOP at 12 months was 14.8 ± 3.9 mmHg, which was significantly lower than both the preoperative medicated IOP of 17.5 ± 3.8 mmHg and the unmedicated IOP of 24.6 ± 3.4 mmHg, and the mean medication burden decreased from 1.49 ± 0.77 to 0.14 ± 0.48. Chen et al. [33] compared phacoemulsification alone with phacoemulsification combined with iStent implantation in eyes with PAC and PACG. At 12 months, postoperative IOP did not differ significantly between the two groups; however, the phacoemulsification combined with iStent implantation group required fewer medications (0.25 ± 0.68 vs. 0.75 ± 1.00) at 12 months. Salimi and coworkers [34] reported significant decreases in the mean IOP (17.6 ± 3.2 to 12.9 ± 2.3 mmHg) and the number of glaucoma medications (2.2 ± 1.2 to 1.2 ± 1.0) in PACG eyes undergoing phacoemulsification combined with iStent implantation and/or GSL. In contrast, Hirooka and colleagues [23] conducted a prospective study of six eyes with PACG treated with phacoemulsification combined with iStent inject W and observed a decrease in mean IOP from 15.3 to 14.2 mmHg at 6 months, which did not reach statistical significance. Issa et al. [37] compared phacoemulsification combined with iStent implantation (phaco-iStent) versus phacoemulsification alone in PACG and failed to find a significant difference in postoperative IOP reduction between groups; however, at 12 months, the phaco-iStent group required fewer glaucoma medications (1.5 ± 1.3 vs. 1.9 ± 1.2) than the phacoemulsification-alone group.

In our study, the mean IOP decreased significantly from 18.3 ± 0.4 mmHg preoperatively to 13.8 ± 0.3 mmHg at 12 months and remained significantly lower than baseline throughout the 12-month follow-up period. The mean number of antiglaucoma medications decreased significantly from 2.85 ± 0.15 at baseline to 2.44 ± 0.13 at 12 months, with significant reductions observed at all postoperative time points, although the magnitudes of reductions were rather smaller compared to the outcomes reported in prior studies and gradually attenuated over time. One may argue that the absolute reduction (−0.48 ± 0.12 at 6 months; −0.41 ± 0.13 at 1 year) in the postoperative medication burden was small in the present study. However, such a small reduction may still be clinically meaningful in the context of our study sample, where about 40% of the enrolled eyes had severe glaucoma and needed to maintain a low target IOP after surgery. The differences between our results and those of prior studies may be related to differences in the clinical characteristics of the study samples, as earlier studies enrolled relatively fewer eyes with advanced glaucoma and more extensive PAS, which could have led to different postoperative IOP and medication outcomes.

### 4.2. PAS Extent, Angle Intervention, and Subgroup Findings

We further examined whether the effects of iStent implantation on IOP and medications varied according to the extent of PAS. When stratified by the PAS extent, eyes with PAS involvement less than 180° of the angle showed a sustained and statistically significant reduction (−4.4 ± 0.4 mmHg at postoperative 12 months) in IOP throughout the entire 12-month follow-up period. In this group, the number of antiglaucoma medications also decreased significantly after surgery and remained statistically significant through 12 months, indicating a sustained reduction in medication burden. In contrast, although the eyes with more extensive PAS involvement of >180° showed significant postoperative IOP improvement (−4.6 ± 1.0 mmHg at postoperative 12 months) at all time points, no significant reduction in the number of antiglaucoma medications was observed at any postoperative time point. Such outcomes, which varied depending on the PAS extent in the present study, may be due to the differences in the surgical approach and the extent of angle intervention. In this study, the angle intervention was performed only in the nasal 180°, with selective GSL and subsequent iStent implantation. Given this surgical approach, eyes with PAS involvement exceeding 180° may have retained some residual PAS. Such residual synechial closure and unaddressed trabecular or distal outflow resistance could have limited functional recovery of the conventional outflow system, thereby attenuating the long-term medication-sparing effect despite significant postoperative IOP reduction. These findings suggest that baseline disease severity and angle status may influence postoperative outcomes, which may partly explain the differences between our results and prior reports.

Compared with our study cohort, prior studies included patients with relatively milder glaucoma severity (−4.2 dB, Chen et al.’s study; −5.9 dB, Salimi et al.’s study) [33,34]. Similarly, in the study by Issa et al. [37], the baseline functional loss was relatively less advanced in the phaco-iStent group (−6.2 dB) than in the phacoemulsification-alone group (−9.3 dB). Intraoperative GSL for PAS was also required in a subset of eyes, occurring in 8.4% of the phaco-iStent group and in 18.9% of the phacoemulsification-alone group, suggesting that differences in baseline disease profile and angle status may influence postoperative medication outcomes. By contrast, our study subjects had more advanced glaucoma at baseline (mean MD, −9.7 ± 8.6 dB), which may reflect greater chronic trabecular and downstream outflow dysfunction and a higher propensity for residual outflow limitation. Moreover, our study included only PACG patients, whereas Hernstadt et al.’s study sample consisted of PAC and PACG [32], which may partly explain variability in medication reduction across the studies. Therefore, the magnitude and pattern of reductions in IOP and medication number observed in our study should be interpreted in the context of a more advanced PACG cohort, which may contribute to differences from prior reports.

### 4.3. Factors Associated with 12-Month Postoperative Outcomes

To further clarify which baseline factors independently influenced long-term IOP and medication outcomes in our study, we performed multivariable analyses at postoperative 12 months. Preoperative IOP was the only variable that showed a significant positive association with IOP reduction at 12 months in both univariable and reduced multivariable analyses. This finding may be explained by the greater potential for postoperative IOP reduction in eyes with higher baseline pressure, along with a larger pressure gradient facilitating aqueous outflow once trabecular resistance is bypassed. In this context, prior studies have reported limited data on prognostic factors in angle-closure eyes undergoing iStent implantation. Previously, Chen and colleagues [33] reported that phacoemulsification alone and thinner preoperative RNFL thickness were associated with failure to achieve complete success, whereas Hernstadt et al. [32] found that higher preoperative medicated IOP was associated with failure to achieve complete success in PAC and PACG eyes undergoing combined phacoemulsification and iStent implantation. They suggested that the elevated baseline IOP may reflect more advanced dysfunction of the conventional outflow pathway, which could limit the effectiveness of trabecular bypass surgery. They further proposed that impaired aqueous flow in the collector channels and aqueous veins may contribute to this association, potentially limiting the outflow capacity despite successful trabecular bypass [32]. However, these theoretical assumptions need to be proven in further studies.

With regard to the medication outcomes in the present study, a greater extent of PAS was associated with a smaller reduction in medications at 12 months. One possible hypothesis is that PAS extent may reflect not only an anatomic finding but also chronic angle-closure–related remodeling or reduced residual function of the conventional outflow pathway. In addition, more extensive PAS may be associated with a greater likelihood of residual or recurrent PAS after surgery. These factors may partly explain the smaller degree of medication reduction observed in eyes with more extensive PAS [14]. Anterior chamber depth was also associated with medication reduction, with greater anterior chamber depth associated with a smaller reduction in medications. One possible explanation is that a shallower anterior chamber may represent greater lens-related anterior segment crowding, in which phacoemulsification may produce greater postoperative deepening of the anterior chamber and widening of the angle [38]. Conversely, in eyes with relatively deeper anterior chambers, the anatomic change after phacoemulsification may be smaller, or non-lens-related mechanisms may contribute more substantially to angle closure, which may be associated with a smaller reduction in medications. Baseline medication burden was consistently associated with medication reduction across models, with eyes on more medications preoperatively tending to undergo greater de-escalation by 12 months. In addition, VFI was positively associated with medication reduction, suggesting that eyes with more preserved visual field function tended to achieve greater postoperative medication de-escalation.

### 4.4. Strengths and Limitations

The present study has several strengths. First, by reporting outcomes of combined phacoemulsification, iStent implantation and GSL in patients with PACG, an area in which real-world evidence remains limited, this study provides additional clinical information for an understudied population. Second, we evaluated the impact of PAS extent on the differences in the surgical response in PACG. Third, by using multivariable analyses to evaluate factors associated with changes in IOP and medications, we found that the preoperative IOP level and anterior segment anatomic factors were associated with the long-term outcomes, thereby providing information that may help predict treatment response.

However, this study also has several limitations. First, due to its retrospective nature, the results may reflect the specific clinical setting and patient demographics of our cohort. The relatively small sample size may limit the generalizability of our findings. Because we included only eyes with at least 12 months of follow-up, selection or attrition bias cannot be excluded. Moreover, the relatively short follow-up period limited the assessment of the long-term durability of surgical outcomes. In addition, postoperative gonioscopy was not performed at standardized time points, and postoperative angle status and PAS recurrence were inconsistently documented; therefore, PAS recurrence and residual PAS after surgery could not be assessed. Second, the independent contribution of each procedure could not be fully disentangled because iStent implantation was performed in combination with phacoemulsification and GSL. The lack of a comparator group limits the interpretation of the extent to which iStent implantation contributed to the observed IOP reduction and changes in glaucoma medication use. Phacoemulsification alone is already known to significantly lower IOP in patients with PACG, and the additional effect of GSL remains controversial. Therefore, the additional IOP-lowering effectiveness of iStent implantation in PACG should be interpreted with caution. Nevertheless, our findings indicate that combined phacoemulsification, iStent implantation, and GSL may provide meaningful reductions in IOP in eyes with PACG, which may help broaden therapeutic options for these patients. Third, GSL was performed only in the nasal 180° (not in the 360°) of the anterior chamber angle in the present study. Therefore, the effect of iStent implantation on IOP and medication burden observed with Phaco-GSL in this study may differ from those where complete GSL has been performed in combination with phacoemulsification. Fourth, because the PAS > 180° subgroup included a relatively small number of eyes, subgroup findings related to PAS extent should be interpreted cautiously. Further studies with larger sample sizes and standardized postoperative gonioscopic evaluation are warranted to investigate the influence of PAS extent on postoperative outcomes. Fifth, heterogeneity in the type and number of iStent devices implanted per eye may have influenced surgical outcomes and should be considered when interpreting the results.

## 5. Conclusions

In conclusion, our findings suggest that phacoemulsification combined with GSL and iStent implantation may be an effective treatment option to lower IOP in PACG patients. However, the reduction in glaucoma medication use was limited in this study cohort. Preoperative IOP was identified as a factor associated with IOP reduction at 12 months. Further prospective comparative studies with a larger sample size are warranted to verify its clinical benefits over phacoemulsification with or without goniosynechialysis in PACG.

## Figures and Tables

**Table 1:** Baseline Demographic and Clinical Characteristics.

Characteristics	Values (%)
Age (years)	75.36 ± 7.04
Male	14 (28.0)
Laterality (Right)	27 (41.5)
HTN	18 (36.0)
DM	13 (26.0)
Prior Laser Procedures	
No	31 (47.7)
LPI	34 (52.3)
Extent of PAS	
90° < PAS ≤ 180°	53 (81.5)
PAS > 180°	12 (18.5)
Preoperative IOP (mmHg)	18.3 ± 2.9
Glaucoma medications (number)	2.85 ± 1.20
Visual Field	
Visual field index (%)	76.29 ± 27.30
Mean deviation (dB)	−9.66 ± 8.57
Pattern standard deviation (dB)	4.91 ± 3.64
RNFL thickness (μm)	74.40 ± 24.36
Biometric parameters	
SE of refractive error (D)	0.70 ± 1.57
Central corneal thickness (μm)	509.52 ± 33.84
White-to-white (mm)	11.57 ± 0.53
Anterior chamber depth (mm)	2.40 ± 0.29
Lens thickness (mm)	5.02 ± 0.37
Axial length (mm)	22.93 ± 0.81
iStent types	
iStent	21 (32.3)
iStent inject	2 (3.1)
iStent inject W	33 (50.8)
iStent infinite	9 (13.8)
Stents implanted per eye	
Two stents	56 (86.2)
Three stents	9 (13.8)

Values are presented as mean ± standard deviation or number (%), unless otherwise indicated. HTN = hypertension; DM = diabetes mellitus; LPI = laser peripheral iridotomy; PAS = peripheral anterior synechiae; IOP = intraocular pressure; RNFL = retinal nerve fiber layer; SE = spherical equivalent.

**Table 2:** Univariable and multivariable linear regression analyses of preoperative factors associated with intraocular pressure reduction at 12 months postoperatively.

	Univariable			Multivariable			
Variable	B	95% CI	*p*-Value	B	95% CI	*p*-Value	VIF
Age	−0.025	−0.151 to 0.100	0.686	0.008	−0.103 to 0.119	0.885	1.049
Axial length	−0.549	−1.401 to 0.303	0.202	—	—	—	—
White-to-white	−0.409	−2.080 to 1.263	0.625	—	—	—	—
ACD	1.186	−1.781 to 4.154	0.426	−0.478	−3.048 to 2.092	0.710	1.100
CCT	0.004	−0.019 to 0.027	0.745	—	—	—	—
Extent of PAS	−0.356	−1.239 to 0.528	0.422	−0.312	−1.022 to 0.398	0.380	1.011
RNFL thickness	−0.001	−0.039 to 0.037	0.971	—	—	—	—
No. of medications	−0.310	−1.031 to 0.410	0.391	—	—	—	—
Preoperative IOP	0.672	0.442 to 0.903	<0.001	0.707	0.443 to 0.970	<0.001	1.077
Lens thickness	0.426	−2.011 to 2.863	0.727	—	—	—	—
VFI	0.007	−0.030 to 0.044	0.702	—	—	—	—
MD	0.043	−0.073 to 0.159	0.459	—	—	—	—
PSD	−0.029	−0.276 to 0.218	0.815	—	—	—	—

Values are presented as regression coefficients (B) with 95% confidence intervals (CIs). The reduced multivariable model included age, ACD, extent of PAS, and preoperative IOP. VIF = variance inflation factor; ACD = anterior chamber depth; CCT = central corneal thickness; PAS = peripheral anterior synechiae; RNFL = retinal nerve fiber layer; IOP = intraocular pressure; VFI = visual field index; MD = mean deviation; PSD = pattern standard deviation.

**Table 3:** Univariable and multivariable linear regression analyses of preoperative factors associated with medication reduction at 12 months postoperatively.

	Univariable			Multivariable			
Variable	B	95% CI	*p*-Value	B	95% CI	*p*-Value	VIF
Age	0.027	−0.017 to 0.071	0.225	—	—	—	—
Axial length	−0.281	−0.576 to 0.015	0.062	—	—	—	—
White-to-white	−0.112	−0.702 to 0.478	0.705	—	—	—	—
ACD	−1.066	−2.076 to −0.056	0.039	−1.150	−1.805 to −0.496	0.001	1.083
CCT	−0.006	−0.014 to 0.001	0.108	—	—	—	—
Extent of PAS	−0.141	−0.268 to −0.014	0.030	−0.122	−0.201 to −0.043	0.003	1.065
RNFL thickness	0.005	−0.008 to 0.019	0.435	—	—	—	—
No. of medications	0.574	0.375 to 0.773	<0.001	0.695	0.545 to 0.845	<0.001	1.056
Preoperative IOP	−0.048	−0.152 to 0.057	0.363	—	—	—	—
Lens thickness	0.704	−0.133 to 1.541	0.097	—	—	—	—
VFI	0.013	0.001 to 0.026	0.038	0.014	0.007 to 0.022	0.001	1.159
MD	0.038	−0.002 to 0.078	0.064	—	—	—	—
PSD	−0.064	−0.150 to 0.022	0.142	—	—	—	—

Values are presented as regression coefficients (B) with 95% confidence intervals (CIs). The reduced multivariable model included the number of medications, ACD, extent of PAS, and VFI. VIF = variance inflation factor; ACD = anterior chamber depth; CCT = central corneal thickness; PAS = peripheral anterior synechiae; RNFL = retinal nerve fiber layer; IOP = intraocular pressure; VFI = visual field index; MD = mean deviation; PSD = pattern standard deviation.

## Data Availability

The data underlying this article will be shared on reasonable request to the corresponding author.

## Abbreviations

The following abbreviations are used in this manuscript:

PACG	primary angle closure glaucoma
IOP	intraocular pressure
POAG	primary open-angle glaucoma
LPI	laser peripheral iridotomy
PAS	peripheral anterior synechiae
GSL	goniosynechialysis
Phaco-GSL	phacoemulsification and goniosynechialysis
MIGS	minimally invasive glaucoma surgery
Phaco-GSL-iStent	phacoemulsification combined with iStent implantation and goniosynechialysis
IRB	Institutional Review Board
ISGEO	International Society of Geographical and Epidemiological Ophthalmology
RNFL	retinal nerve fiber layer
BCVA	best-corrected visual acuity
VFI	visual field index
PSD	pattern standard deviation
MD	mean deviation
HTN	hypertension
DM	diabetes mellitus
SE	spherical equivalent
